# Quantitative evaluation of LED‐based optical autofocus module

**DOI:** 10.1111/jmi.70148

**Published:** 2026-07-29

**Authors:** S. Habte, S. Kumar, J. Lightley, E. Garcia, M. A. A. Neil, P. M. W. French

**Affiliations:** ^1^ Physics Department Imperial College London London UK; ^2^ Francis Crick Institute London UK

**Keywords:** autofocus, led filament, microscope, microscopy, optics, standard deviation, superluminescent diode

## Abstract

We report an improved version of the open‐source optical autofocus (OAF) module (*openAF*) for light microscopy, replacing the superluminescent diode (SLD) with a multimode‐optical fibre‐coupled LED as a more accessible AF module light source. We also present a method for independently quantifying AF system performance using 2D autocorrelation analysis of astigmatic fluorescent nanobead images. We applied this method to evaluate both this new LED‐based *openAF* module and our previous SLD‐based module implemented on a microscope with a 100 × 1.4 numerical aperture oil‐immersion objective lens. Our new approach compensates for AF light source power variation, and we demonstrate that the LED‐based system can provide axial stability with a standard deviation below 10 nm for at least 45 min from a cold start as the LED power varies, whereas it reaches thermal equilibrium.

## 1 INTRODUCTION

Autofocus (AF) techniques are essential in optical microscopy to ensure that the sample remains in focus despite mechanical or environmental perturbations such as thermal drift and/or variations in distance from objective lens to coverslip. AF is a basic requirement of automated microscopy, for example, for high‐content analysis (HCA) and whole‐slide scanning—noting that throughput is an increasing priority for light microscopy, for example, for automated pathology or for acquiring large experimental data sets for training machine learning models.^1^, ^2^

As well as high throughput or long timescale imaging at high numerical aperture (NA), for example, for time‐lapse assays, AF is also important for super‐resolution microscopy. Single molecule localisation microscopy (SMLM)^3^, ^4^, ^5^ can achieve nanometre‐scale resolution but requires data acquisitions of many minutes, during which the sample can drift in three dimensions. Although lateral drift can be corrected post‐acquisition using spatial cross‐correlation of image data or fiduciary markers,^6^, ^7^ axial drift by more than the depth of field (DOF) can cause point spread function (PSF) blurring and thus localisation errors that degrade reconstructed super‐resolved images. For 3D SMLM, which may be implemented using methods such as astigmatism,^8^ biplane^9^ and engineered PSFs^10^ that provide axial localisation via the calibrated variation of the PSF, the axial resolution can also be compromised by drift in focus.

AF can be implemented using existing motorised microscope functionality with software‐based approaches, or by the use of additional optical hardware modules for optical AF (OAF). Software‐based AF techniques typically rely on image quality metrics of the sample image—such as sharpness, entropy, spatial frequency content^11^, ^12^, ^13^—or image file size,^14^ that are evaluated across an axial image *z*‐stack to determine best focus. However, this *z*‐stack typically needs to be acquired for each different sample or field of view (FOV)—significantly slowing image data acquisition rate for slide scanning or multiwell plate assays. Moreover, such image metric‐based approaches work best with thin samples and may not lock to a specific plane in thick samples such as tissue slices or 3D cell cultures, or with samples that evolve over time.

OAF approaches utilise an optical beam (often in the infrared spectral region to minimise both phototoxicity and overlap with fluorescence signals) that is reflected from one of the microscope coverslip interfaces during imaging. Defocus can be quantified via position or image‐based metrics of reflected AF light detected using a photodiode array or a camera, such as AF beam's intensity, displacement or size.^15^, ^16^, ^17^ These approaches also entail the acquisition of a ‘calibration’ AF *z*‐stack data set to which the test value of the defocus metric can be compared. However, this is usually required only once in an imaging session (for a specific microscope configuration) and generally does not need to be repeated for each different sample or FOV. Informed by the calculated defocus, the sample or objective lens can be continuously moved to bring the sample back to focus. OAF can provide faster operation for real‐time ‘closed loop’ operation and extended axial range compared to image‐based methods, and it can operate with reduced phototoxicity as the AF is independent of the primary imaging process and may utilise low‐power infrared radiation.

A hybrid AF approach is to use features in the sample^18^ or fiduciary markers^19^, ^20^ in the real‐time images, such as sub‐resolution polystyrene beads or gold nanorods affixed to the coverslip, to enable simultaneous lateral and axial position locking or 3D drift‐correction using either the primary imaging detector or a separate AF imaging system. However, this approach requires additional sample preparation and/or complex computational analysis to realise 3D drift‐correction, which may include fitting, for example,^21^ or deep learning, for example,^22^ to account for field‐dependent optical aberrations.

Although several OAF configurations have been reported, for example, reviewed in,^23^ including AF capabilities built into commercial microscopes,^24^, ^25^ there is a paucity of robust AF modules available for after‐market implementation on custom‐built or commercial microscopes. With the growing number of custom microscopes being developed for diverse applications, there is a need for robust, adaptable and cost‐effective OAF modules, including open‐source approaches, that offer straightforward implementation and flexibility. Thus, the development of robust OAF implementations remains an active field of research, noting that even integrated commercial AF systems can fail with challenging samples or specialised experimental requirements. One consideration is that thermal or mechanical drifts in the AF systems themselves can also impact imaging performance without being detected or corrected. A further challenge is that OAF can be challenging to implement with high‐NA oil immersion objective lenses when imaging biological samples. This is because the back‐reflected AF signal's intensity depends on the ∼0.4% Fresnel reflection at the coverslip/aqueous sample interface, potentially making this signal weaker than unwanted back‐reflections from other surfaces in the optical train or light backscattered from the biological sample itself. Thus, a practical AF implementation needs to be robust against drift in the AF system itself and against unwanted back‐reflections and other spurious signals incident on the AF detector.

To develop a robust OAF, it is important to quantity the performance independently of the OAF system, preferably with a rapid and convenient method. In previous work from our laboratory,^16^, ^26^ we analysed *z*‐stacks of fluorescent beads (using the software *PSFj* ^27^) to determine the net defocus after operation of our OAF, as well as used the sharpness of a transmitted light image of a USAF test chart as a readout of defocus. However, the repeated acquisition of bead stacks to provide a defocus metric was time‐consuming, and the use of a USAF test chart provided an anomalously strong back reflection of the AF beam that may not recapitulate the performance experienced when imaging biological samples. More recently, an astigmatism‐based OAF utilising a laser diode, *PiFocus*,^17^ was reported that used the astigmatic 3D localisation method of a bead sample to characterise time‐lapse performance of their OAF—demonstrating precision below 10 nm.

In this paper, we review our development of OAF modules for *openScopes*,^28^ a modular, sustainable and open‐source microscope platform designed to enable rapid prototyping by instrument developers and to widen access to research‐grade microscopy for users in lower resourced settings. These open‐source OAF modules are designed to be compatible with most fluorescence microscopes, including for SMLM, HCA and slide‐scanning, and are specifically designed to work with *openFrame*‐based instruments,^26^ which can support diverse imaging modalities, including automated SMLM based on *easySTORM* ^16^, ^29^ and quantitative phase imaging using single‐shot *pDPC*.^30^, ^31^

Our first OAF module implementation^16^ utilised a single‐mode laser diode collimated by a spherical lens and a rectangular slit to provide an AF beam. When focused on the microscope coverslip interface, this beam exhibited different orthogonal confocal parameters. The back‐reflected beam was then focused onto a CMOS camera with the aim of using the beam size to provide the defocus metric, as we expected this to be more robust against drifts in alignment that could impact metrics based on AF beam position. This approach enables the OAF to provide higher resolution when considering the beam projection parallel to the slit (as the beam diameter at pupil of objective lens is larger) and longer range when considering the perpendicular beam projection that underfills the objective lens pupil. Because the AF camera image data could be compromised by interference between the AF signal and (unwanted) axially and time‐varying back‐reflections, we used a convolutional neural network (CNN) trained on calibration AF camera *z*‐stacks^16^ acquired over multiple days to determine sign and magnitude of defocus from camera image data, rather than using an analytic algorithm. This CNN‐based OAF approach achieved ±100 µm operation range with an accuracy better than the DOF of the 1.4 NA objective lens with a two‐step algorithm.^16^ However, obtaining the composite CNN training data was time‐consuming, with data acquired over ∼10 days to account for drifts in the alignment of the AF system itself. Furthermore, the training data *z*‐stacks needed to be sampled to the desired precision of the OAF module, which entailed large data volumes. Although this CNN‐based OAF module worked reliably for automated (multiwell plate) SMLM assays requiring several hours of image data acquisition and proved reliable over >12 months, the time‐consuming requirement to acquire new training data for each specific microscope configuration was a practical disadvantage.

To overcome these challenges, we developed an analytical approach to determine defocus from AF camera image data that we described as *openAF* ^26^ For this system we replaced the combination of spherical collimation and a slit with astigmatic collimation of the light from a single‐mode‐optical fibre (SMOF) using two orthogonal cylindrical lenses (CLs) of different focal lengths. One of these CLs is slightly translated from the collimation position to provide a shift in the focal plane at the AF camera, which enables the sign of defocus to be determined from a single AF camera test image. Furthermore, we replaced the AF laser diode with a super luminescent diode (SLD), significantly reducing interference of the AF signal with spurious reflections such that the background was not coherent with the AF beam and did not vary with defocus. As a result, the same background image could be subtracted from each AF camera image. Defocus was determined using the width of the Fourier transform of the average image intensity projection of the recorded AF camera image along each orthogonal axis.^26^ This *openAF* module was implemented on an *openFrame*‐based (*easySTORM*) microscope^26^ to realise single‐shot closed‐loop operation over an axial range of ±37 µm with an accuracy better than 200 nm (as measured using bead *z*‐stacks). In two‐step AF mode, the axial range could be extended up to ±68 µm. Although the *openAF* module provides a robust OAF capability that has been used for SMLM, including for super‐resolved CLEM,^32^ the SLD is a relatively expensive and fragile component, and both SLDs and laser diodes present laser safety concerns that must be carefully managed.

Here, we report further development of the *openAF* module where the SMOF‐coupled SLD has been replaced with a low‐cost multimode‐optical fibre (MMOF)‐coupled LED—with other elements of the *openAF* module remaining the same. As well as being easier and cheaper to acquire, the use of an LED light source removes laser safety concerns during OAF module installation and operation. To quantify the performance of this new OAF module, we implemented astigmatic fluorescence imaging on the *openFrame*‐based microscope by inserting a CL in the fluorescence imaging path, as is commonly used for 3D SMLM. To provide rapid and straightforward quantification of its performance, we developed a novel image autocorrelation‐based software tool to compute the defocus independently of the OAF readout and applied this to time‐lapse (astigmatic) image data of fluorescent nanobeads. As this method works with a single fluorescence bead image, it is simpler to implement than acquiring bead *z*‐stack data to determine defocus, and the computational analysis is much faster than 3D (SMLM software‐based) localisation of individual beads. When characterising our LED‐based *openAF* module, we observed an LED power‐dependent artefact that was apparent as the LED reached thermal equilibrium after being switched on. We, therefore, modified the *openAF* algorithm to include a normalisation of the detected AF light intensity and subsequently achieved a precision better than 10 nm from switch‐on over extended time‐lapse measurements (∼45 min).

During this work, we also developed an ‘infinity alignment tool’ that provides a simple means to optimise focusing of the imaging arm (tube lens to camera) of a microscope, including where astigmatic imaging has been implemented.

## 2 METHODS

### 2.1 LED‐based *openAF*

**FIGURE 1:**
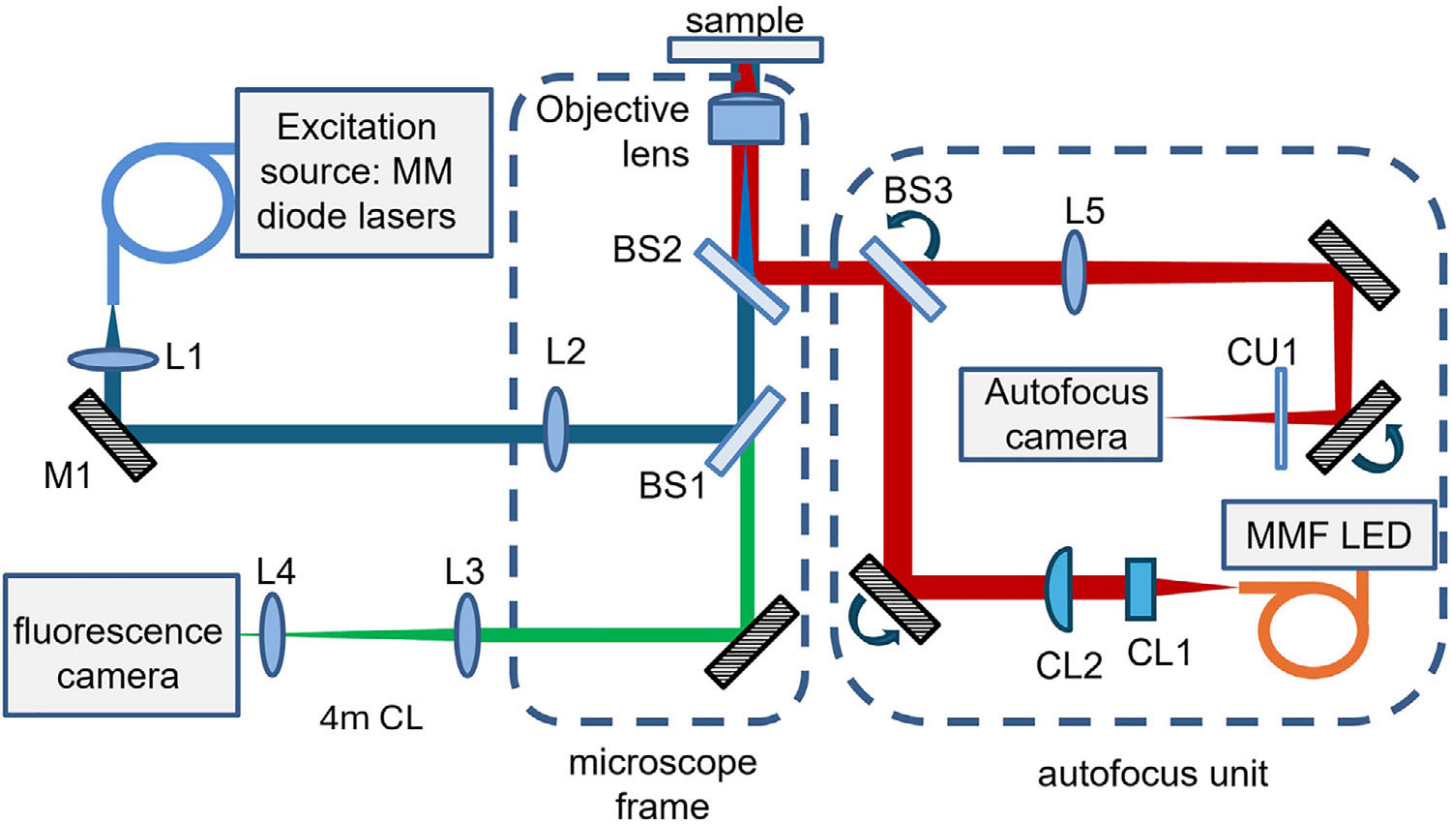
Schematic of openFrame‐based microscope with LED‐based optical autofocus: Excitation source—multimode diode laser bank (Triline, Cairn Research Ltd), L1—60 mm collimating lens (AC254‐060‐A, Thorlabs), L2—200 mm focusing lens (49‐364, Edmund Optics), BS1—multiline dichroic (ZT405_465_625_825rpc‐UF2, Chroma), objective lens—100 × 1.4 NA oil objective lens (UPLSAPO) mounted on a piezo‐electric actuator (Piezo *z*‐stage Q‐545.140 with controller E‐873.1AT, PI) to prove axial translation, L3—200 mm tube lens (MXA20696, Nikon), 4 m focal length cylindrical lens (4000 YO 65, Comar Optics, Ltd), L4 (0.35× Motic c‐mount adapter (1101001904111, Motic), camera (CellCam Kikker, Cairn Research Ltd). Autofocus unit: 850 nm LED (Thorlabs M850F1 with LEDD1B driver) coupled into 50 µm core diameter multimode‐optical fibre (MMOF) (Thorlabs M14L), CL1—6.4 mm focal length cylindrical lens (Thorlabs LJ1227L1‐B, Thorlabs), CL2—20 mm focal length cylindrical lens (LJ1960L1‐B, Thorlabs), L5—200 mm focal length lens in autofocus unit (AC254‐200‐B, Thorlabs), BS2—800 nm short pass autofocus dichroic (69‐220 Edmund Optics), BS3—50:50 beamsplitter (47‐026 Edmund Optics), CU1—cleanup filter (FF01‐842/56‐25, Semrock), autofocus camera (CM3‐U3‐31S4M‐CS, FLIR).

Figure 1 presents a schematic of the modified *openAF* AF module coupled to the *openFrame*‐based microscope.^26^ Here, the SLD has been replaced with an 850 nm LED (Thorlabs M850F1, with LEDD1B driver) coupled into a 50 µm core diameter MMOF (Thorlabs M14L). This extended optical source with low spatial coherence is not as well‐collimated as the single spatial mode SLD beam coupled via an SMOF and the resulting image on the AF camera includes light emerging from the MMOF cladding as well as the core. There is also significant unwanted scattered light within the AF module, and so we introduce a 3D printed 3 × 10 mm^2^ rectangular aperture in front of the collimating CLs to block unwanted radiation emerging from the cladding. Video S1 shows how the AF camera image varies as the microscope coverslip is scanned through focus and Figure S1A shows AF camera images acquired when the sample coverslip is at defocus values of −1, 0 and +1 µm. For comparison, Figure S1B shows analogous AF camera images when the SLD/SMOF is used. The OAF calculates the microscope defocus from the spatial profiles of these images, comparing the derived AF image metrics to those for a reference calibration data set that can be acquired within a few minutes at the start of each imaging session. These calibration data are obtained by determining the defocus metric value for each background‐subtracted AF camera image in a *z*‐stack of images acquired at known stage displacements from the focal plane and interpolating them to produce smooth curves.

**FIGURE 2:**
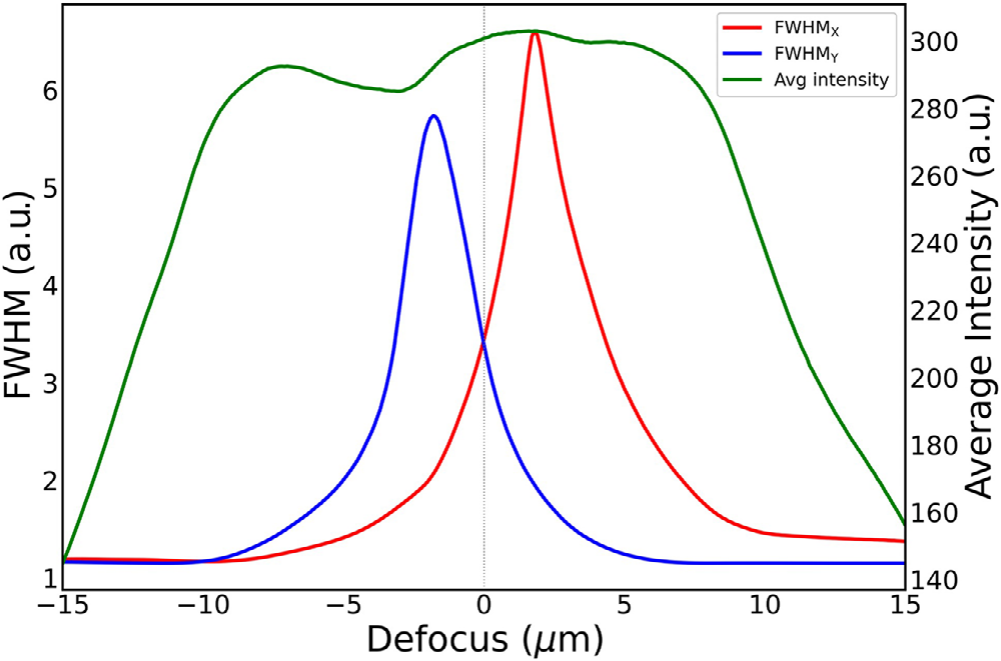
LED openAF axial calibration curves acquired between ±15 µm: interpolated plots of FWHM *X* (red) and FWHM *Y* (blue) of the Fourier transforms of the average image intensity projections along *Y* and *X*, respectively, with the average intensity (green).

Figure 2 shows the defocus calibration curves of three defocus metrics calculated from a background‐subtracted AF camera *z*‐stack acquired as the 100 × 1.4 NA oil immersion objective lens (Olympus UPLSAPO) was translated through focus from −15 to +15 µm: the variation of the average AF camera image intensity (green) and the full width at half maximum (FWHM) of the Fourier transforms of the average image intensity projections of the recorded signal for each orthogonal axis (red and blue). These ‘calibration’ AF *z*‐stack image data were acquired in steps of 100 nm together with a background image recorded with the objective lens translated to 50 µm below focus. The calibration data acquisition took 2 min for this ±15 µm range. In operation, the *openAF µManager* plug‐in AF module continuously acquires AF camera images, from which the background image is subtracted before computing the defocus metrics and comparing them to the values in the calibration look‐up table. In closed‐loop operation the FWHM *X* (parallel to the axis of the longer focal length CL2) metric is used to calculate the two possible defocus values (either side of the peak) through cubic spline interpolation of adjacent values, and the corresponding FWHM *Y* is used to determine the true defocus value. In the cases where the defocus would be near (within ∼±2 µm) the maxima of the FWHM *X* and FWHM *Y* curves, the orthogonal FWHM metric is used to determine defocus. The *µManager* AF plug‐in^26^ then moves the objective lens to correct the calculated defocus.

The average camera intensity decreases as the image of the LED radiation emerging from the MMOF becomes out of focus. Because the quasi‐collimated LED/MMOF beam diverges much more rapidly than the collimated SLD/SMOF beam, the total operating range of the LED/MMOF AF is lower—at ∼±15 µm range. This is adequate for time‐lapse imaging at a given FOV, and the AF can be operated in closed loop over this range. Typically, the AF camera is set to acquire images with an integration time of ∼100 ms, and the total time to acquire each image, calculate the defocus and send the correction command to the objective lens *z*‐stage is ∼250 ms.

### 2.2 Measurement of autofocus performance over time using astigmatic imaging with autocorrelation analysis

To quantify the performance of this LED/MMOF‐based *openAF* implementation and compare it with the previously published SLD/SMOF implementation, we measured the microscope's defocus over time independently of the AF system. This was undertaken by acquiring time‐lapse images of 100 nm diameter fluorescent beads (TetraSpeck, T7279) with a CL of 4 m focal length inserted between the fluorescence imaging tube lens and the fluorescence camera assembly—which included a 0.3× Motic demagnifier mounted directly onto the (CellCam Kikker, Cairn Research Ltd) camera in order to permit imaging at a higher frame rate.^26^ This astigmatic imaging would enable each fluorescent bead to be axially localised following the usual 3D SMLM approach.^33^ However, since we are only interested in a single defocus value for the fluorescent bead image, rather than localisation of individual fluorescent beads, we computed the 2D autocorrelation function of the bead images across the FOV through the Wiener–Khinchin theorem, as illustrated in Figure S2. We derived a defocus metric based on the ratio of the widths of Gaussian functions fitted to the orthogonal (*x* and *y*) mean intensity projections of the central peak of the 2D autocorrelation function (Figure S2C). The protocol to acquire the calibration data for this astigmatic imaging defocus model is given in the Supporting Information.

**FIGURE 3:**
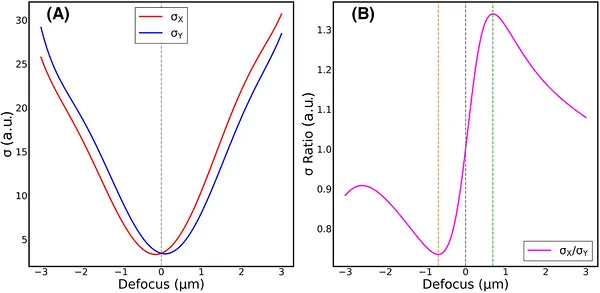
Astigmatic autocorrelation defocus model: (A) *σ_X_*_,_ *_Y_* of the 1D Gaussian fit to mean intensity projections in *X* (red) and *Y* (blue) of the central autocorrelation function peak; (B) ratio *σ_X_*/*σ_Y_* of Gaussian fits to *Y* and *X* mean projections of the central peak of 2D autocorrelation function where the interpolated monotonic range between the extrema (∼±0.7 µm range) is used for direct estimation of defocus value.

Videos S2 and S3 show the *z*‐stack of acquired fluorescent bead images and of the corresponding maximum peak of the 2D autocorrelation function, respectively. Figure S2 shows exemplar plots of the maximum of the 2D autocorrelation functions at different values of defocus with the mean intensity projections along *X* and *Y*. Figure 3A shows the standard deviation, *σ*, of the Gaussian fits to the mean intensity projections along *X* (red) and *Y* (blue) of the central autocorrelation peak as a function of defocus, and Figure 3B; the ratio *σ_X_*/*σ_Y_*. The latter provides a monotonic range for precise interpolation and direct look‐up table of defocus from the autocorrelation function of a given fluorescent image within the range of approximately ± 0.7 µm, defined between the extrema of the ratio plots. Thus, this analysis of astigmatic fluorescence images of a fluorescent bead sample provides a rapid means to independently quantify defocus of the microscope image and evaluate the performance of (any) AF module.

### 2.3 Infinity alignment tool

We note that this characterisation of the performance of the OAF requires careful alignment and focusing of the fluorescence and AF imaging systems. To independently determine the conditions of good focus for each system, we developed the ‘infinity alignment tool’ shown in Figure S5. This comprises a metal tube with a graticule (R1DS3N2, Thorlabs) fixed at the focal plane of a 4× magnification objective lens RMS4×, Thorlabs) that could be screwed directly into the microscope objective holder (replacing the primary microscope objective lens) and so present an image at infinity to the tube lens. The fluorescence imaging camera can then be set to the position corresponding to the sharpest image of the graticule (illuminated with a convenient diffuse light source), and the primary microscope objective lens can then be replaced and translated to locate the (fluorescent bead) sample in the focal plane. The same infinity alignment tool can also be used to set up the separation of the AF tube lens L5 and the AF camera.

To precisely set the graticule in focal plane of the 4× objective lens in the infinity alignment tool, we use a shearing interferometer (SI100, Thorlabs) to check the collimation of an alignment laser beam that is retroreflected from the graticule surface after passing through the 4× objective lens—a procedure that is only required once. We believe this alignment tool is generally useful to rapidly set up any light microscope. For astigmatic imaging systems, such as we implement here and which are often used in 3D SMLM microscopes, the cross at the centre of the graticule can be used to determine the camera position that brings each axis of the astigmatic imaging system into focus—and then the camera can be set equidistant between them, at the circle of least confusion—as can be confirmed by observing equal defocussing of the graticule ruling in each orthogonal direction.

## 3 RESULTS

**FIGURE 4:**
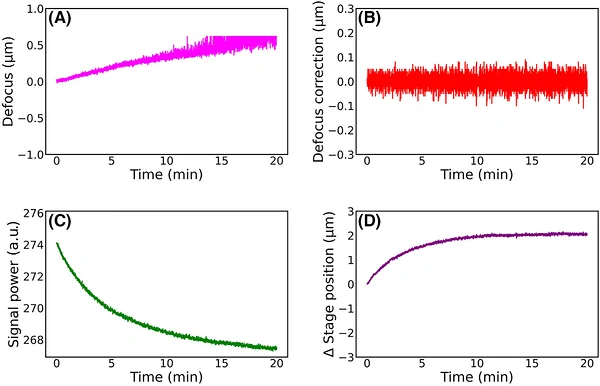
Time‐lapse plots during operation of the LED/multimode‐optical fibre (MMOF) openAF autofocus module switched on from cold, revealing a correlation between LED power and focus drift apparent only by astigmatic imaging. (A) The defocus (µm) measured by astigmatic nanobead imaging (magenta), showing >500 nm drift before exceeding the astigmatic imaging range after 20 min; (B) the required defocus correction (µm) reported by the LED/MMOF openAF module (red); (C) variation in LED power (a.u.) represented by the average intensity of the autofocus camera image (green), showing a ∼1.5% decrease over the 20‐min warm‐up period; and (D) the position (µm) of the piezo‐electric *z*‐stage reported by its encoder (purple).

The astigmatic fluorescent bead imaging method was first used to monitor the accuracy of the LED *openAF* AF module where the LED was coupled into a 50 µm core diameter MMOF and the sample of 100 nm diameter fluorescent beads was imaged with 100 × 1.4 NA oil immersion objective lens (UPLSAPO). We observed that, although the AF generally functioned well, there could be a significant drift in focus after the LED was switched on. This behaviour is illustrated in Figure 4, which presents the time‐lapse plots of (A) the defocus measured with the astigmatic bead imaging (magenta), (B) the defocus correction applied by the LED/MMOF *openAF* module (red), (C) the variation in power of the LED as represented by the average intensity at the AF camera (green) and (D) the position of the piezo‐electric‐actuated *z*‐stage reported by its encoder (purple). It is apparent from (C) that the LED power decreased by ∼2% during the ∼20‐min warm‐up period after switching on, and this led to a drift in the image focal plane of >500 nm before exceeding the range of the astigmatic imaging after 20 min. However, this focus drift caused by the LED power change is not detected by the *openAF* as reported by the required defocus correction (B). This figure illustrates the importance of independently measuring the imaging defocus to quantify the performance of an OAF system during operation, rather than relying solely on the OAF readout.

We reasoned that there was a systematic power‐dependent error in the calculation of the *openAF* defocus metric that was due to the background signal changing with LED power—noting that the subtraction of the pre‐recorded background image was the only intensity‐dependent component of the *openAF* calculation of defocus. We, therefore, compared each real‐time AF camera image with a reference in‐focus image in the AF calibration *z*‐stack and used the average intensities to continuously scale the pre‐recorded background intensity image before subtracting it from the (real‐time) AF camera image being used to compute defocus. This effectively normalises the background subtraction to variations in LED power.

**FIGURE 5:**
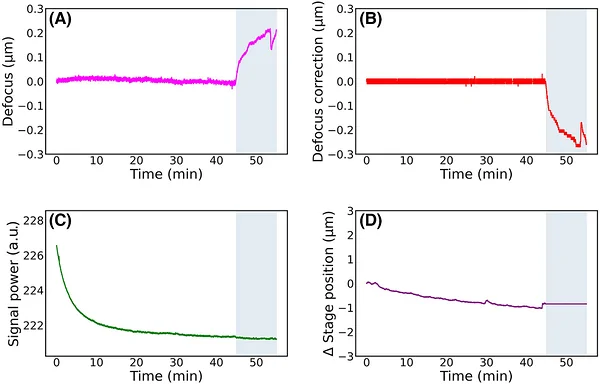
Time‐lapse plots during the operation of the LED/multimode‐optical fibre (MMOF) openAF autofocus module switched on from cold with continuous background intensity normalisation: (A) Defocus (µm) measured with the astigmatic nanobead imaging (magenta), showing SD of 7 nm over 45 min; (B) the required defocus correction (µm) reported by the LED/MMOF openAF module (red); (C) variation in LED power (a.u.) as represented by the average intensity at the autofocus camera (green), showing ∼2% decrease over the 45 min period; and (D) position (µm) of the piezo‐electric *z*‐stage reported by its encoder (purple). Note that the *z*‐stage was disabled at 45 min.

As is evident in Figure 5, this normalisation of the background image efficiently eliminated the LED power‐dependent error, providing robust operation of the AF immediately after the LED was switched on, despite the LED power changing by 2% during the first 30 min. In this mode the AF accurately maintained focus with a standard deviation of ∼7 nm over 45 min. At this point, the signal to the *z*‐stage was switched off such that the astigmatic defocus measurement—and the *openAF* defocus measurement—reported the focal drift of the uncorrected microscope. This performance will ultimately be limited by the precision of the piezo *z*‐stage (PI, Q‐545.140) with controller (PI, E‐873.1AT), which has a specified minimum displacement of 6 nm and an encoder resolution of 1 nm, as well as by the precision of the astigmatic imaging measurement.

**FIGURE 6:**
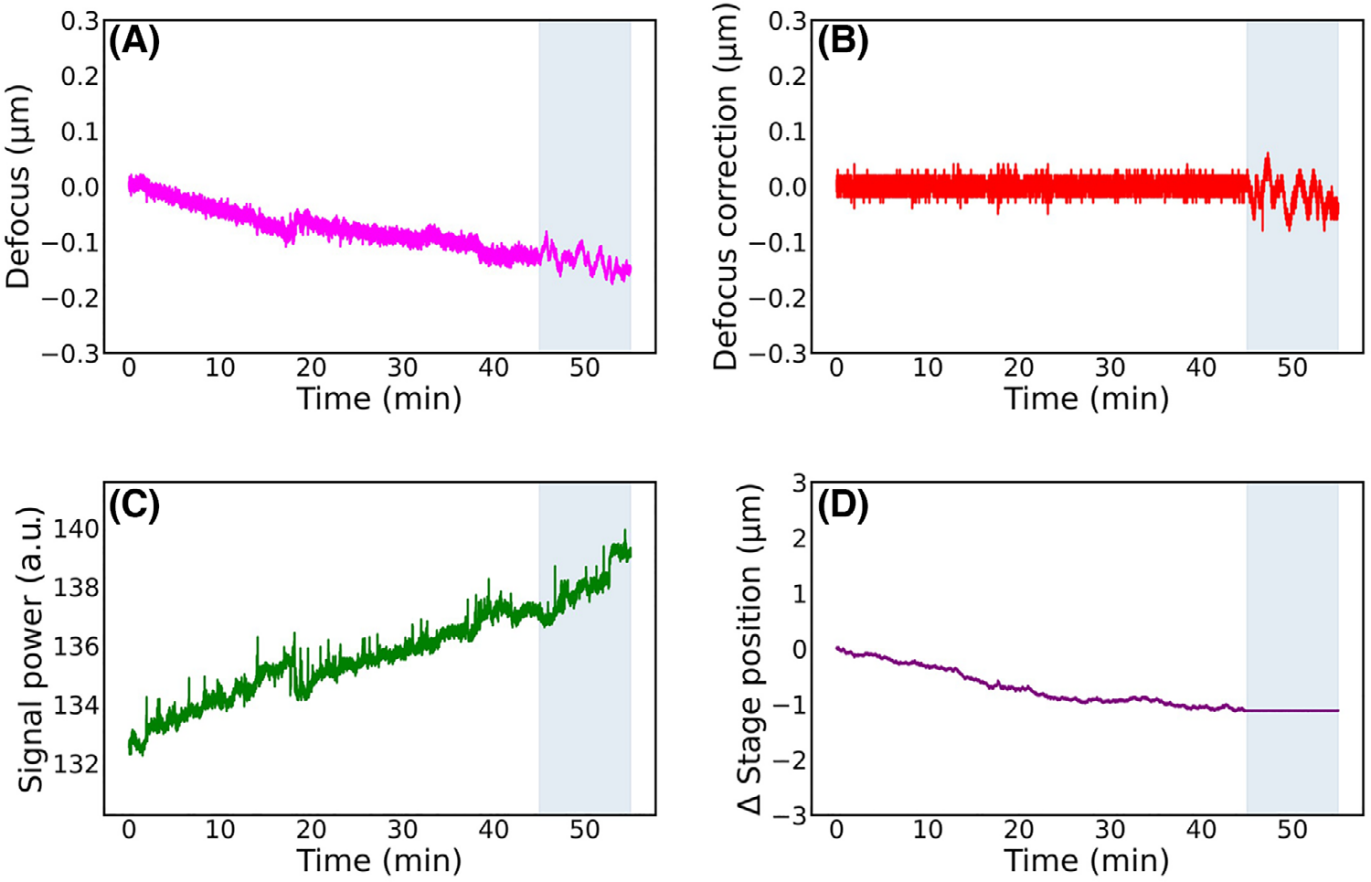
Time‐lapse plots of the operation of the superluminescent diode (SLD)/single‐mode‐optical fibre (SMOF) openAF autofocus module switched on from cold without continuous background intensity normalisation: (A) The defocus (µm) measured with the astigmatic nanobead imaging (magenta), showing drift of >100 nm over 45 min; (B) the required defocus correction (µm) reported by the SLD/SMOF openAF module (red); (C) the variation in power (a.u.) of the SLD as represented by the average intensity of the autofocus camera image (green), showing an increase of ∼5% over the 45 min; and (D) the position (µm) of the piezo‐electric *z*‐stage reported by its encoder (purple). Note that the *z*‐stage was disabled at 45 min.

**FIGURE 7:**
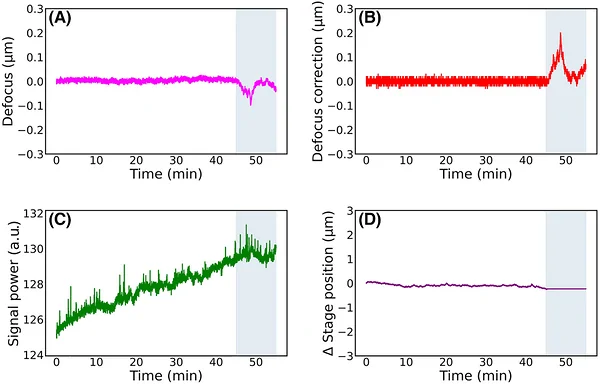
Time‐lapse plots of the operation of the superluminescent diode (SLD)/single‐mode‐optical fibre (SMOF) openAF autofocus module switched on from cold with continuous background intensity normalisation: (A) The defocus (µm) measured with the astigmatic nanobead imaging (magenta), showing SD of 8 nm; (B) the defocus correction (µm) reported by the SLD/SMOF openAF module (red); (C) the variation in SLD power (a.u.) as represented by the average intensity at the autofocus camera image (green); and (D) the position (µm) of the piezo‐electric *z*‐stage reported by its encoder (purple). Note that the *z*‐stage was disabled at 45 min.

We applied the same astigmatic fluorescent bead imaging approach to monitor the precision of the *openAF* AF module configured with the SLD/SMOF light source. Figure 6 shows how the SLD power at the AF camera varied over time, with a ∼5% decrease in power leading to a defocus drift >100 nm—which is lower than for the (unnormalised) LED‐based OAF. However, the performance was still improved with the implementation of background intensity normalisation before subtraction, as illustrated in Figure 7, where the focus was maintained with a standard deviation of ∼8 nm over 45 min, until the signal to the *z*‐stage was switched off.

**FIGURE 8:**
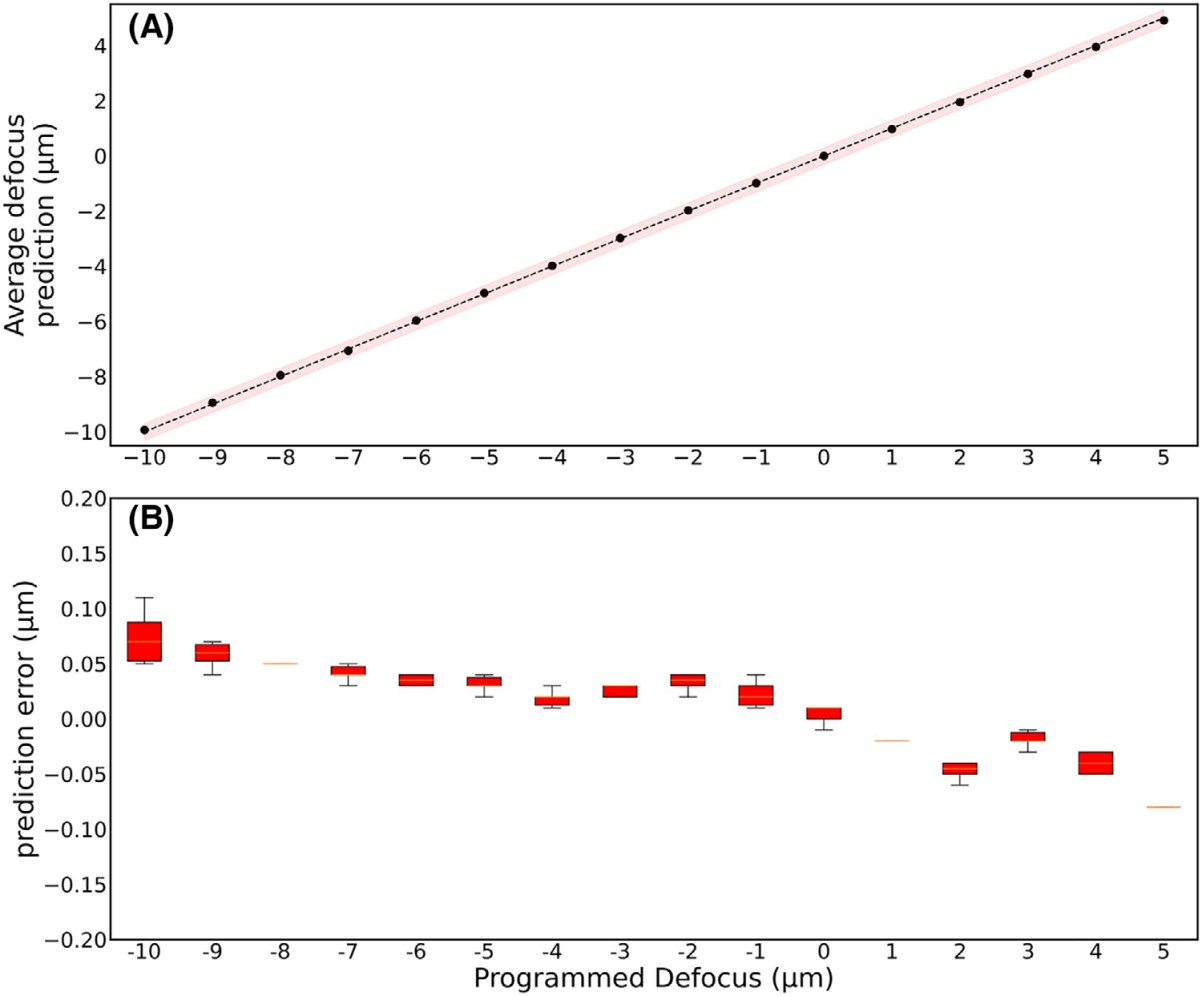
Autofocus range measurement: (A) the average defocus prediction (µm) versus programmed (‘true’) defocus for a 15 µm range from −10 µm to +5 µm showing that the average prediction remains within the depth of field across the full range; (B) boxplot of prediction errors (µm) versus programmed (‘true’) defocus.

To quantify the operating range of the LED/MMOF openAF AF system, we implemented a programme to translate the objective lens to predetermined values of defocus, with 10 repeated acquisitions at each value. Figure 8A shows a plot of the programmed (‘true’) defocus against the average defocus value calculated by the *openAF* AF module. This indicated the system's operational range to be 15 µm, with the range being asymmetric about the focus position due to the focal planes of the AF camera image and the fluorescence camera image being offset. Figure 8B shows a box plot presenting the median and interquartile range of the defocus error for each programmed objective lens displacement. It is apparent that this uncertainty in the predicted defocus is significantly less than the DOF of the 100 × 1.4 NA oil objective lens (UPLSAPO) (200 nm for a wavelength of 550 nm).

The LED/MMOF *openAF* operating range is limited by the rapid expansion of the LED radiation emerging from the MMOF with defocus, which overfills the AF camera sensor. When defocus correction required exceeds 15 µm range, which may occur for applications such as multiwell plate imaging, the *openAF* system could employ a two‐step correction method, as previously reported^26^, utilising the variation of average intensity at the AF camera over the range of the calibration data for the first step defocus metric.

## 4 CONCLUSIONS

In conclusion, we have presented a straightforward and robust means to quantify the performance of AF systems that is based on astigmatic imaging of fluorescent beads analysed using a 2D autocorrelation‐based algorithm. We demonstrated this using the open‐source *openAF* AF module implemented on an *openFrame*‐based microscope with a 100 × 1.4 NA oil immersion objective lens. We quantify the performance of a new LED/MMOF implementation that offers convenience at significantly lower cost, and without raising laser safety concerns. We have also quantified the performance of the previously published SLD/SMOF configuration.

The accuracy of the astigmatic imaging method to quantify defocus revealed that small variations in the power of the AF light source could degrade the performance of the AF module. We showed that this could be mitigated by continuously normalising the average intensity of the calibration background image before subtraction from the real‐time AF camera images, enabling the new LED/MMOF implementation to provide SD of <10 nm axial stability over at least 45 min. We note that variations in the average intensity at the AF camera could arise from changes in the coupling efficiency of light coupled into the SMOF/MMOF, as well as in the emitted power from the LED/SLD.

To further demonstrate the utility of this LED/MMOF OAF approach, we observed the accuracy over a 3‐h time‐lapse with sparser temporal sampling to reduce photobleaching of the fluorescent nanobeads. This performance is presented in Figure S3. We also implemented this LED/MMOF OAF approach with a 40× air objective and present the time‐lapse performance in Figure S4. As the depth of field of the objective lens is increased, this reduces the precision of both the AF module and the image autocorrelation‐based readout of the fluorescent beads. Nevertheless, the observed precision is far below the imaging DOF.

We believe that this improved LED/MMOF‐based *openAF* AF module will be useful for SMLM and time‐lapse microscopy, noting that it performs well when imaging biological samples with a high‐NA oil immersion objective lens that provides the lowest reflectivity (∼0.4%) from the coverslip/aqueous medium interface of OAF systems.

The LED/MMOF *openAF* closed‐loop operating range may be extended beyond 15 µm with further optimisation of the system, for example, by changing the collimation of the MMOF—noting, however, that using an MMOF with a smaller core diameter would result in less power available at the AF camera and therefore a longer AF response time, and/or by reducing the focal length of the AF tube lens. Alternatively, the calibration data range can be extended, and the average AF camera intensity can be used to provide the first step in a two‐step AF method. These parameters will be explored in future work.

We have also presented a simple ‘infinity alignment tool’ that is useful for rapidly aligning conventional and astigmatic imaging paths in light microscopes.

## DISCLOSURE

For the purpose of open access, the authors have applied a CC BY public copyright licence to this submitted manuscript.

## Supporting information



Supporting Information

Supporting Information

Supporting Information

Supporting Information

## Data Availability

The data that support the findings of this study are openly available in Quantitative evaluation of LED‐based optical AF module at https://doi.org/10.5281/zenodo.19349029. The modified *openAF* software is available at https://github.com/imperial‐photonics/openAF. The autocorrelation analysis software is available at https://github.com/sara98habte/autofocus‐evaluation.

## References

[1] Chen, P. H. C. , Gadepalli, K. , MacDonald, R. , Liu, Y. , Kadowaki, S. , Nagpal, K. , Kohlberger, T. , Dean, J. , Corrado, G. S. , Hipp, J. D. , Mermel, C. H. , & Stumpe, M. C. (2019). An augmented reality microscope with real‐time artificial intelligence integration for cancer diagnosis. Nature Medicine, 25, 1453–1457. 10.3390/life15040654 31406351

[2] Tsuneki, M. (2025). Deep learning approaches for pathological image classification. Journal of Oral Biosciences, 67, 100696. 10.1016/j.job.2025.100696 41274680

[3] Rust, M. , Bates, M. , & Zhuang, X. (2006). Sub‐diffraction‐limit imaging by stochastic optical reconstruction microscopy (STORM). Nature Methods, 3, 793–796. 10.1038/nmeth929 16896339 PMC2700296

[4] Heilemann, M. , van de Linde, S. , Schüttpelz, M. , Kasper, R. , Seefeldt, B. , Mukherjee, A. , Tinnefeld, P. , & Sauer, M. (2008). Subdiffraction‐resolution fluorescence imaging with conventional fluorescent probes. Angewandte Chemie International Edition, 47, 6172–6176. 10.1002/anie.200802376 18646237

[5] Fölling, J. , Bossi, M. , Bock, H. , Medda, R. , Wurm, C. A. , Hein, B. , Jakobs, S. , Eggeling, C. , & Hell, S. W. (2008). Fluorescence nanoscopy by ground‐state depletion and single‐molecule return. Nature Methods, 5, 943–945. 10.1038/nmeth.1257 18794861

[6] Wang, Y. , Schnitzbauer, J. , Hu, Z. , Li, X. , Cheng, Y. , Huang, Z. L. , & Huang, B. (2014). Localization events‐based sample drift correction for localization microscopy with redundant cross‐correlation algorithm. Optics Express, 22, 15982–15991. 10.1364/OE.22.015982 24977854 PMC4162368

[7] Schnitzbauer, J. , Strauss, M. T. , Schlichthaerle, T. , Schueder, F. , & Jungmann, R. (2017). Super‐resolution microscopy with DNA‐PAINT. Nature Protocols, 12, 1198–1228. 10.1038/nprot.2017.024 28518172

[8] Huang, B. , Wang, W. , Bates, M. , & Zhuang, X. (2008). Three‐dimensional super‐resolution imaging by stochastic optical reconstruction microscopy. Science, 319, 810–813. 10.1126/science.1153529 18174397 PMC2633023

[9] Juette, M. F. , Gould, T. J. , Lessard, M. D. , Mlodzianoski, M. J. , Nagpure, B. S. , Bennett, B. T. , Hess, S. T. , & Bewersdorf, J. (2008). Three‐dimensional sub‐100 nm resolution fluorescence microscopy of thick samples. Nature Methods, 5, 527–529. 10.1038/nmeth.1211 18469823

[10] Pavani, S. R. , Thompson, M. A. , Biteen, J. S. , Lord, S. J. , Liu, N. , Twieg, R. J. , Piestun, R. , & Moerner, W. E. (2009). Three‐dimensional, single‐molecule fluorescence imaging beyond the diffraction limit by using a double‐helix point spread function. Proceedings of the National Academy of Sciences of the United States of America, 106, 2995–2999. 10.1073/pnas.0900245106 19211795 PMC2651341

[11] Sun, Y. , Duthaler, S. , & Nelson, B. J. (2004). Autofocusing in computer microscopy: Selecting the optimal focus algorithm. Microscopy Research and Technique, 65, 139–149. 10.1002/jemt.20118 15605419

[12] Zhou, W. , & Yang, D. (2023). Analysis and comparison of automatic image focusing algorithms in digital image processing. Journal of Radiation Research and Applied Sciences, 16, 100672. 10.1016/j.jrras.2023.100672

[13] Liao, J. , Bian, L. , Bian, Z. , Zhang, Z. , Patel, C. , Hoshino, K. , Eldar, Y. C. , & Zheng, G. (2016). Single‐frame rapid autofocusing for brightfield and fluorescence whole slide imaging. Biomedical Optics Express, 7, 4763–4768. 10.1364/BOE.7.004763 27896014 PMC5119614

[14] Knapper, J. , Collins, J. T. , Stirling, J. , McDermott, S. , Wadsworth, W. , & Bowman, R. W. (2022). Fast, high‐precision autofocus on a motorised microscope: Automating blood sample imaging on the OpenFlexure microscope. Journal of Microscopy, 285, 29–39. 10.1111/jmi.13064 34625963

[15] Liu, C. S. , Hu , P.‐H. , Wang , Y.‐H. , Ke , S.‐S. , Lin , Y.‐C. , & Chang , Y.‐H. (2010). Novel fast laser‐based auto‐focusing microscope. IEEE Sensors, 481–485. 10.1109/ICSENS.2010.5690153

[16] Lightley, J. , Görlitz, F. , Kumar, S. , Kalita, R. , Kolbeinsson, A. , Garcia, E. , Alexandrov, Y. , Bousgouni, V. , Wysoczanski, R. , Barnes, P. , Donnelly, L. , Bakal, C. , Dunsby, C. , Neil, M. A. A. , Flaxman, S. , & French, P. M. W. (2022). Robust deep learning optical autofocus system applied to automated multiwell plate single molecule localization microscopy. Journal of Microscopy, 288, 130–141. 10.1111/jmi.13020 34089183

[17] Rahmani, A. , Cox, T. , Achary, A. T. A. , & Ponjavic, A. (2024). Astigmatism‐based active focus stabilisation with universal objective lens compatibility, extended operating range and nanometer precision. Optics Express, 32, 13331–13341. 10.1364/OE.520845 38859306

[18] Silvestri, L. , Müllenbroich, M. C. , Costantini, I. , Di Giovanna, A. P. , Mazzamuto, G. , Franceschini, A. , Kutra, D. , Kreshuk, A. , Checcucci, C. , Toresano, L. O. , Frasconi, P. , Sacconi, L. , & Pavone, F. S. (2021). Universal autofocus for quantitative volumetric microscopy of whole mouse brains. Nature Methods, 18, 953–958. 10.1038/s41592-021-01208-1 34312564

[19] Coelho, S. , Baek, J. , Graus, M. S. , Halstead, J. M. , Nicovich, P. R. , Feher, K. , Gandhi, H. , Gooding, J. J. , & Gaus, K. (2020). Ultraprecise single‐molecule localization microscopy enables in situ distance measurements in intact cells. Science Advances, 6, eaay8271. 10.1126/sciadv.aay8271 32494604 PMC7164934

[20] Bon, P. , Bourg, N. , Lécart, S. , Monneret, S. , Fort, E. , Wenger, J. , & Lévêque‐Fort, S. (2015). Three‐dimensional nanometre localization of nanoparticles to enhance super‐resolution microscopy. Nature Communications, 6, 7764. 10.1038/ncomms8764 PMC4525210 26212705

[21] Li, Y. , Mund, M. , Hoess, P. , Deschamps, J. , Matti, U. , Nijmeijer, B. , Sabinina, V. J. , Ellenberg, J. , Schoen, I. , & Ries, J. (2018). Real‐time 3D single‐molecule localization using experimental point spread functions. Nature Methods, 15, 367–369. 10.1038/nmeth.4661 29630062 PMC6009849

[22] Fu, S. , Shi, W. , Luo, T. , He, Y. , Zhou, L. , Yang, J. , Yang, Z. , Liu, J. , Liu, X. , Guo, Z. , Yang, C. , Liu, C. , Huang, Z.‐L. , Ries, J. , Zhang, M. , Xi, P. , Jin, D. , & Li, Y. (2023). Field‐dependent deep learning enables high‐throughput whole‐cell 3D super‐resolution imaging. Nature Methods, 20, 459–468. 10.1038/s41592-023-01775-5 36823335

[23] Z hang, Y. , Liu , L. , Gong , W. , Yu , H. , Wang , W. , Zhao , C. , Wang , P. , & Ueda , T. (2018). Autofocus system and evaluation methodologies: A literature review. Sensors and Materials, 30, 1165–1174. 10.18494/SAM.2018.1785

[24] Nikon, N. , Perfect focus system . https://www.nikonusa.com/fileuploads/pdfs/Instruments/TE2000_PFS.pdf

[25] Zeiss, Z. , Definite focus . https://www.imdik.pan.pl/images/Usługi_dokumenty/Przydatne_informacje/2d_Definite_Focus_information.pdf

[26] Lightley, J. , Kumar, S. , Lim, M. Q. , Garcia, E. , Görlitz, F. , Alexandrov, Y. , Parrado, T. , Hollick, C. , Steele, E. , Roßmann, K. , Graham, J. , Broichhagen, J. , McNeish, I. A. , Roufosse, C. A. , Neil, M. A. A. , Dunsby, C. , & French, P. M. W. (2023). openFrame: A modular, sustainable, open microscopy platform with single‐shot, dual‐axis optical autofocus module providing high precision and long range of operation. Journal of Microscopy, 292, 64–77. 10.1111/jmi.13219 37616077 PMC10953376

[27] Theer, P. , Mongis, C. , & Knop, M. (2014). PSFj: Know your fluorescence microscope. Nature Methods, 11, 981–982. 10.1038/nmeth.3102 25264772

[28] www.openscopes.com

[29] Kwakwa, K. , Savell, A. , Davies, T. , Munro, I. , Parrinello, S. , Purbhoo, M. A. , Dunsby, C. , Neil, M. A. , & French, P. M. (2016). easySTORM: A robust, lower‐cost approach to localisation and TIRF microscopy. Journal of Biophotonics, 9, 948–957. 10.1002/jbio.201500324 27592533

[30] Kalita, R. , Flanagan, W. , Lightley, J. , Kumar, S. , Alexandrov, Y. , Garcia, E. , Hintze, M. , Barkoulas, M. , Dunsby, C. , & French, P. M. W. (2021). Single‐shot phase contrast microscopy using polarisation‐resolved differential phase contrast. Journal of Biophotonics, 14, e202100144. 10.1002/jbio.202100144 34390220

[31] Liu, H. , Kumar, S. , Garcia, E. , Flanagan, W. , Lightley, J. , Dunsby, C. , & French, P. M. W. (2024). Open‐source implementation of polarisation‐resolved single‐shot differential phase contrast microscopy (pDPC) on a modular openFrame‐based microscope. HardwareX, 21, e00622. 10.1016/j.ohx.2024.e00622 39877828 PMC11773044

[32] Lumkwana, D. , Lightley, J. , Vesga, A. G. , Folter, J. , Domart, M.‐C. , Maclachlan, C. , Kumar, S. , Evans, J. , Peddie, C. , Burrell, A. , Yoshimura, A. , Mangali, A. , Vahrjmeijer, N. , Bhaga, M. , Smit, C. , Titze, B. , Horrocks, M. , Straker, L. C. , Garcia, E. , & Collinson, L. (2025). VP‐CLEM‐Kit: An accessible pipeline for visual proteomics using super resolution volume correlative light and electron microscopy (SR‐vCLEM). BioRxiv, 10.1101/2025.04.08.647770

[33] Huang, B. , Wang, W. , Bates, M. , & Zhuang, X. (2008). Three‐dimensional super‐resolution imaging by stochastic optical reconstruction microscopy. Science, 319, 810–813. 10.1126/science.1153529 18174397 PMC2633023

